# Accelerating fringe projection profilometry to 100k fps at high-resolution using deep learning

**DOI:** 10.1038/s41377-025-01802-4

**Published:** 2025-03-27

**Authors:** Jie Xu, Jindong Tian

**Affiliations:** 1 College of Physics and Optoelectronic Engineering, Shenzhen University, Shenzhen, 518060 China; 2 Guangdong Laboratory of Artificial Intelligence and Digital Economy (Shenzhen), Shenzhen, 518107 China

**Keywords:** Imaging and sensing, Optics and photonics

## Abstract

Fringe projection profilometry, a powerful technique for three-dimensional (3D) imaging and measurement, has been revolutionized by deep learning, achieving speeds of up to 100,000 frames per second (fps) while preserving high-resolution. This advancement expands its applications to high-speed transient scenarios, opening new possibilities for ultrafast 3D measurements.

Fringe projection profilometry (FPP) is a widely adopted three-dimensional (3D) imaging technique with extensive applications in various industrial processes including additive manufacturing, semiconductor inspection, and others^1–4^. It is known for its non-contact nature, high precision, flexibility, and ability to perform full-field measurements. The fundamental principle of FPP is straightforward, relying on a well-defined pinhole model of the imaging system and utilizing triangulation to achieve 3D measurements^5^. This principle has been extensively employed in various 3D measurement techniques^6–8^. What sets FPP apart is its ability to provide high-precision, full-field 3D measurements by effectively utilizing the phase information embedded in periodic grayscale fringe patterns (e.g., sinusoidal fringes). By analyzing the deformation of the fringes on the object’s surface, the phase distribution can be accurately extracted, and the object’s topography can be further reconstructed through proper calibrations.

Although the principle of FPP is relatively simple, its practical performance is constrained by limitations in physical devices and algorithms. These constraints lead to one of FPP’s fundamental challenges: the trade-off between resolution and speed. For example, achieving higher measurement accuracy often requires capturing multiple images with varying frequencies and phase shifts, which inherently restricts the applicability of FPP in dynamic scenarios. While advancements such as faster projectors and high-speed cameras with higher frame rates can mitigate this issue, increasing the camera’s frame rate typically results in shorter exposure times, thereby lowering the signal-to-noise ratio (SNR) and ultimately compromising the accuracy and resolution of 3D measurements. In recent years, continuous breakthroughs in deep learning methods have enabled numerous successful applications in the field of 3D imaging, while also injecting fresh momentum into the development of FPP^9–11^. These advancements hold great potential for overcoming the longstanding trade-off between spatial resolution and temporal performance in current FPP methods.

A recent study published in Light: Science & Applications by the Smart Computational Imaging Laboratory (SCILab) at Nanjing University of Science and Technology introduced a novel ultrafast single-shot super-resolved FPP (SSSR-FPP) approach enabled by deep learning^12^. As shown in Fig. 1, this method leverages two high-speed cameras set at different angles of view to provide the absolute phase information of the measured surface, while maintaining adjustable focal lengths, frame rates, and region-of-interest sizes. By employing convolutional neural networks trained on experimental data, SSSR-FPP effectively maps low-resolution (LR, 160 × 160 pixels) and low-SNR raw fringe images to high-resolution (HR, 480 × 480 pixels) and high-SNR absolute phase maps. This innovative approach enables the generation of high-quality 3D images even at ultra-high frame rates of up to 100,000 frames per second (fps), marking a significant step forward for ultrafast 3D imaging.

**Fig. 1:**
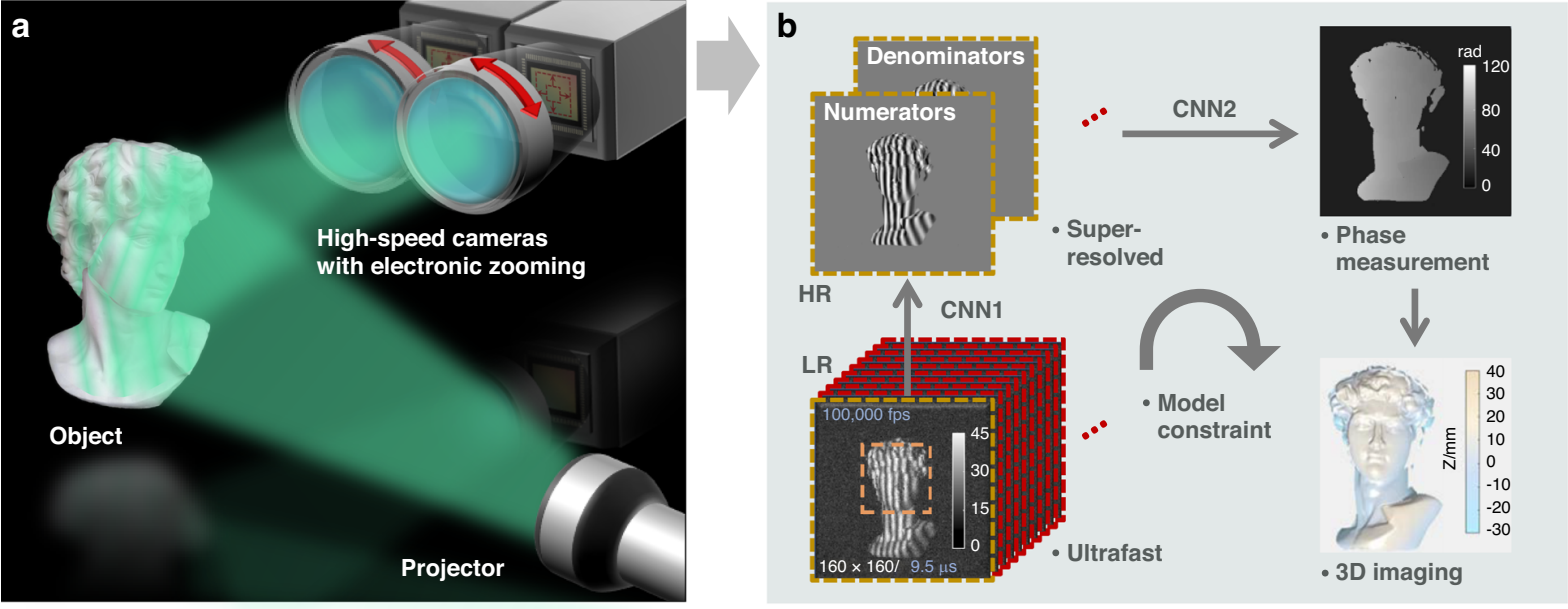
Schematic diagram and workflow of the SSSR-FPP system. **a** The SSSR-FPP system employs two high-speed cameras with adjustable focal lengths, frame rates, and region-of-interest sizes, aiming to generate datasets of mapping LR to HR. **b** The workflow of the proposed SSSR-FPP method consists of two main steps, each utilizing a convolutional neural network (CNN1 and CNN2) with identical architectures but different input and output configurations. Overall, the workflow is model-constrained, maintaining ultrafast acquisition and super-resolution, while also providing the 3D reconstruction algorithm with good adaptability and generalization

The entire process of SSSR-FPP is divided into two steps, each utilizing a CNN with identical architectures but distinct input and output configurations. In the first step, the initial neural network (CNN1) generates HR numerator (sine) and denominator (cosine) terms of the wrapped phase function from the captured LR raw images. These two terms are then used for calculating the HR phase maps through an arctangent function. Since the range of the arctangent function is limited to (−π, π), the resulting phase map is naturally wrapped within a 2π range. To overcome this limitation, SSSR-FPP utilizes a second neural network (CNN2) in the second step, specifically designed to unwrap the phase and accurately map the wrapped phase to the absolute phase with by leveraging the reference surface information. To validate the effectiveness of SSSR-FPP, the researchers conducted experiments on various dynamic targets, demonstrating that the SSSR-FPP technique is capable of achieving HR and ultrafast 3D imaging.

For a typical high-speed imaging system designed for macro scenarios, spatial resolution is primarily constrained by the Nyquist sampling criterion, determined by the pixel size. Recognizing this limitation, the SSSR-FPP framework employs CNN1 trained on experimental data to establish a more realistic mapping relationship from LR to HR. Unlike simulated data or data generated through direct down-sampling methods, the experimental data-driven neural network enables the SSSR-FPP framework to extract richer information with “physically meaningful” prior knowledge of the image formation process. This is crucial for achieving highly accurate phase reconstruction in subsequent stages. Moreover, instead of using a single end-to-end CNN to directly output the phase map, SSSR-FPP divides the process into two distinct steps that incorporate the fundamental principles of FPP. This approach effectively addresses the “domain mismatch” issue and the “black box” problem inherent in deep learning, enhancing the stability and robustness of the reconstruction algorithm under challenging conditions.

Looking ahead, while SSSR-FPP marks a significant breakthrough by pushing FPP’s speed into the 100k fps range, it does not imply that FPP has reached its ultimate potential. Emerging industries, such as real-time 3D modeling for virtual and augmented reality, and online process monitoring in the rapidly evolving field of ultrafast manufacturing, are placing higher demands on 3D imaging. To meet the growing need for faster and more robust detection, future advancements in FPP can be pursued from two perspectives: hardware and methodologies, as illustrated in Fig. 2. In terms of high-speed hardware, novel detection devices—such as event cameras—show great promise in overcoming the limitations of current imaging technologies. With a temporal resolution in the microsecond range, event cameras outperform conventional grayscale cameras by several orders of magnitude. In recent years, these devices have been explored for 3D dynamic measurement applications^13–16^. Methodologies, such as computational imaging techniques based on compressive sensing, have already achieved significant breakthroughs in recording speeds for transient imaging, surpassing 10^8^ fps^17,18^. Furthermore, artificial intelligence technologies leveraging deep neural networks are demonstrating increasingly powerful data processing capabilities, opening new avenues for addressing the inherent spatial-temporal trade-off problem and enabling single-shot reconstructions^19,20^.

**Fig. 2:**
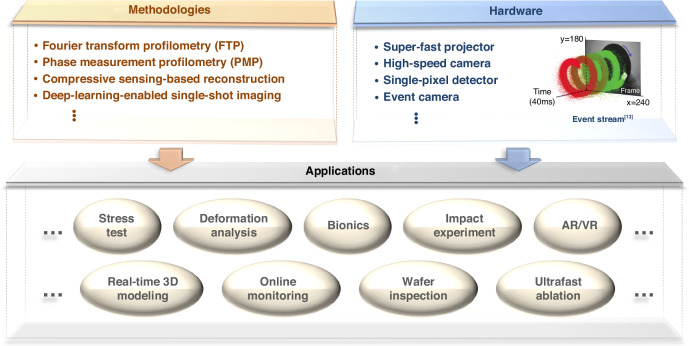
Breakthroughs in ultrafast imaging across methodologies and hardware, and their potential application trends in dynamic and transient 3D scenarios

These advancements provide valuable insights and inspiration for the development of ultrafast 3D imaging technologies. We are confident that, in the near future, the integration of more advanced technologies and innovative methodologies will further push the speed boundaries of FPP measurement, enabling even greater performance and applicability across a wider range of transient scenarios requiring higher frame rates.
